# R.E.N.A.L. nephrometry score predicts postoperative recurrence of localized renal cell carcinoma treated by radical nephrectomy

**DOI:** 10.1007/s10147-015-0879-3

**Published:** 2015-07-29

**Authors:** Akira Nagahara, Motohide Uemura, Atsunari Kawashima, Takeshi Ujike, Kazutoshi Fujita, Yasushi Miyagawa, Norio Nonomura

**Affiliations:** Department of Urology, Osaka University Graduate School of Medicine, 2-2 Yamadaoka, Suita, Osaka 565-0871 Japan

**Keywords:** Imaging, Nephrometry score, Radical nephrectomy, Recurrence, Renal cell carcinoma

## Abstract

**Background:**

We investigated the association between the R.E.N.A.L. nephrometry score (RNS) and the postoperative recurrence of localized renal cell carcinoma (RCC).

**Methods:**

We retrospectively analyzed a database comprising 91 patients with non-small localized RCC (pT1b–T2b) treated by radical nephrectomy at our hospital from January 2002 to March 2010. RNS was scored based on imaging findings at diagnosis. The Cox proportional hazards model was used to predict recurrence-free survival (RFS) and to calculate hazard ratio (HR).

**Results:**

The median age at operation was 63 years (range, 30–85 years). Postoperative recurrence occurred in 19 patients (21 %). Median RNS sum was 9 (range, 5–11). High RNS sum (10–12) was significantly associated with RFS (*P* = 0.0012). Multivariate analysis revealed that high RNS sum [HR, 9.05; 95 % confidence interval (CI), 2.11–63.9; *P* = 0.0019] were significantly associated with RFS. Regarding each component of RNS, only the L component, which referred to tumor location relative to the polar line, was associated with RFS (HR, 15.0; 95 % CI, 2.68–396; *P* = 0.0006).

**Conclusions:**

RNS was associated with RFS in cases of non-small localized RCC (pT1b–2b), thus supporting its utility as a prognostic factor.

## Introduction

Recently, three nephrometry scoring systems, namely, the R. E. N. A. L. nephrometry score (RNS) [1], PADUA score [2], and C-index [3], all of which are based on the anatomical features of renal tumors, were developed to assess the risk of nephron-sparing surgery for a small renal mass including renal cell carcinoma (RCC). These metrics have been validated to predict perioperative complications for partial nephrectomy in many previous studies.

RNS characterizes tumors on the basis of the anatomical features of renal masses on computed tomography (CT) or magnetic resonance imaging (MRI) [1]. Of these scoring systems, only the RNS was associated with histological features of tumor aggressiveness in some reports [4–7]. Accordingly, RNS is expected to be a useful predictor of postoperative recurrence in patients with localized RCC. However, to our knowledge, there has been only one report regarding the association between RNS and postoperative recurrence [8]. For these reasons, in the present study, we decided to use RNS to analyze the association between anatomical features and postoperative recurrence after radical nephrectomy in patients with localized RCC (T1b–T2b).

## Patients and methods

### Patients

We retrospectively analyzed a database comprising 91 consecutive patients with localized RCC (pT1b–2b) treated by radical nephrectomy between January 2002 and March 2013 at the Osaka University Hospital. Staging of RCC was performed according to the 7th edition of TNM staging proposed by the International Union for Cancer Control and the American Joint Committee on Cancer [9]. RNS (www.nephrometry.com) consists of R, E, N, A, and L components based on the following anatomical features of RCC: size, endophytic degree, proximity to the collecting system or renal sinus, position (anterior or posterior), and location relative to the polar line, respectively. If the tumor is in contact with the main renal artery or vein, the suffix h is assigned. RNS was calculated on the basis of findings of preoperative CT or MRI. Recurrence-free survival (RFS) time was calculated from the date of surgery until the date of recurrence or the date of the patient’s last follow-up visit. The study was approved by the institutional review board of Osaka University hospital (approval number: 11397-2).

### Statistical analysis

RFS rate was calculated using the Kaplan–Meier method, and comparisons were made using the log-rank test. The median value of each RNS parameter was used for the cutoff value. For preoperative serum C-reactive protein (CRP) level, 0.2 mg/dl was used for cutoff value. Univariate and multivariate analyses were performed using the COX proportional hazards model to predict RFS and calculate hazard ratio. Variables entered into the model for RFS analysis included patient age, gender, preoperative serum CRP level, histological grade, histological type, microscopic vascular involvement, RNS sum, and each component of RNS. The variables that had a *P* value <0.1 in the univariate analysis were entered into the multivariate analysis. Two kinds of multivariate analysis, one including the RNS sum and the other including each score-constituent parameter of RNS, were performed. All statistical analyses were performed using JMP Pro version 11 (SAS Institute, Tokyo, Japan), with *P* < 0.05 considered as statistically significant.

## Results

The clinical and pathological characteristics of the 91 patients with localized RCC and their treatment outcomes are shown in Table 1. The median age at operation was 63 years (range, 30–85 years). The histological type of RCC of 80 patients was clear cell carcinoma and the remaining 11 were non-clear cell carcinoma. Postoperative recurrence occurred in 19 patients (21 %), and the median time to cancer recurrence after radical nephrectomy was 27 months (range, 1–79 months). The recurrence sites were as follows: lung (10 patients, 53 %), contralateral kidney (2 patients, 11 %), bone (1 patient, 5 %), contralateral ureter (1 patient, 5 %), gallbladder (1 patient, 5 %), pancreas (1 patient, 5 %), small intestine (1 patient, 5 %), hilar lymph node (1 patient, 5 %), and mediastinal lymph node (1 patient, 5 %). In addition, 3 patients (16 %) with cancer recurrence died of RCC progression during the observation periods. The median follow-up period was 65 months (range, 1–156 months). Anatomic characteristics based on RNS are shown in Table 2.

**Table 1:** Clinicopathological characteristics of the 91 patients

Characteristics	Total	(*n* = 91)
Age, years (median)	30–85 (63)	
Gender		
Male	59	(65 %)
Female	32	(35 %)
R/L		
Right	44	(48 %)
Left	47	(52 %)
Preoperative serum CRP level		
≤0.2 mg/dl	72	(79 %)
>0.2 mg/dl	18	(20 %)
Unknown	1	(1 %)
pT stage		
1b	65	(71 %)
2a	18	(20 %)
2b	8	(9 %)
Histological type		
Clear cell carcinoma	80	(88 %)
Non-clear cell carcinoma	11	(12 %)
Histological grade		
1	5	(5 %)
2	73	(80 %)
3	13	(14 %)
Microscopic vascular involvement		
Absent	82	(90 %)
Present	5	(5 %)
Unknown	4	(4 %)
Recurrence		
No	72	(79 %)
Yes	19	(21 %)
Follow up, months (median)	1–156 (65)

**Table 2:** Anatomic characteristics as based on R.E.N.A.L. nephrometry score

Characteristics	Total	(*n* = 91)
R.E.N.A.L. nephrometry score sum		
4–6	2	(2 %)
7–9	44	(48 %)
10–12	45	(49 %)
R component		
1	0	(0 %)
2	60	(66 %)
3	31	(34 %)
E component		
1	33	(36 %)
2	53	(58 %)
3	5	(5 %)
N component		
1	2	(2 %)
2	2	(2 %)
3	87	(96 %)
A component		
a	22	(24 %)
p	17	(19 %)
x	52	(57 %)
L component		
1	15	(16 %)
2	29	(32 %)
3	47	(52 %)
Presence of h		
h (−)	49	(54 %)
h (+)	42	(46 %)

**Fig. 1:**
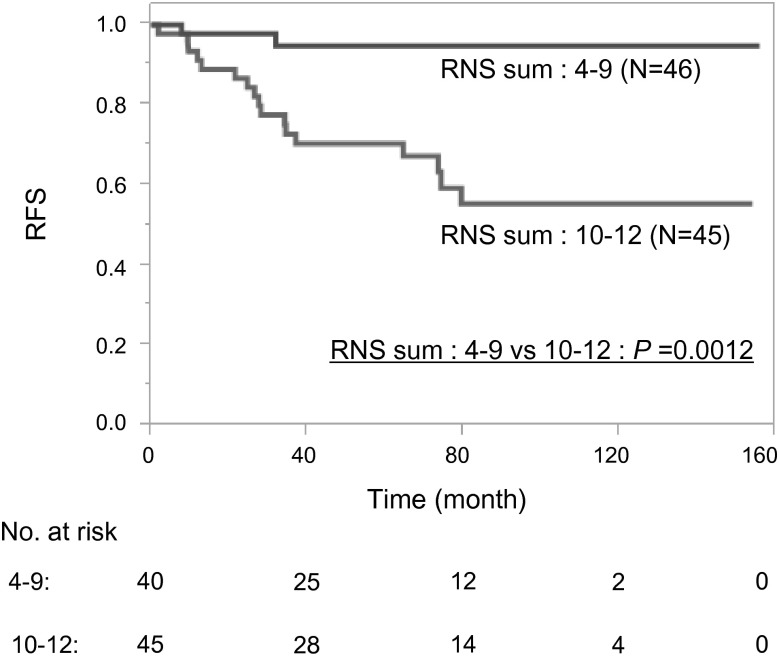
Probability estimates of recurrence-free survival (*RFS*) rate in 91 patients in two groups based on R.E.N.A.L. nephrometry score (*RNS*) sum (4–9 vs. 10–12)

**Fig. 2:**
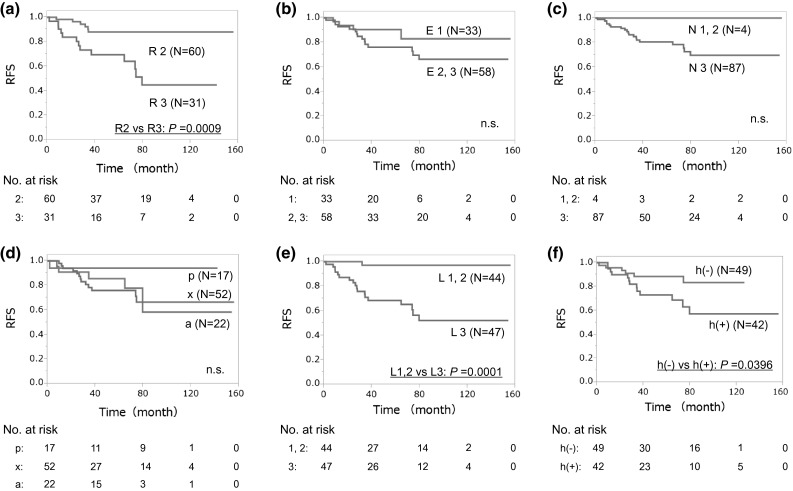
Probability estimates of recurrence-free survival rate (*RFS*) in 91 patients stratified based on R component (score: 2 vs. 3) (**a**), E component (score: 1 vs. 2, 3) (**b**), N component (score: 1, 2 vs. 3) (**c**), A component (a vs. p vs. x) (**d**), L component (score: 1, 2 vs. 3) (**e**), and presence of h (absent vs. present) (**f**)

Figure 1 shows the Kaplan–Meier curve of RFS rate stratified according to RNS sum (4–9 vs. 10–12). RFS rate in patients with a low RNS sum was much better than that in patients with a high RNS sum (*P* = 0.0012). The 5- and 10-year RFS rates in patients with high RNS sum (70 % and 55 %, respectively) were significantly worse than those in patients with low RNS sum (95 % and 95 %, respectively). Figure 2 shows the Kaplan–Meier curve of the RFS rate stratified according to each component of RNS. RFS rate was significantly different when stratified by the R component (*P* = 0.0009), the L component (*P* = 0.0001), and the presence of hilar tumor (*P* = 0.0396).

The results of univariate analysis are shown in Table 3. Of several factors examined, RNS sum, R component, L component, and the presence of h were significant factors in predicting postoperative cancer recurrence. Age at operation, preoperative serum CRP level and A component (x vs. p) were weakly associated with RFS rate on univariate analysis. The multivariate analysis including age at operation, preoperative serum CRP level, RNS sum, A component, and presence of h showed that only the RNS sum (HR, 9.05; 95 % CI, 2.11–63.9; *P* = 0.0019) was an independent predictive factor for postoperative cancer recurrence (Table 3). A second multivariate analysis replacing RNS sum with the R and L components, which were significant score-constituent factors of RNS on univariate analysis, showed that the L component (HR, 15.0; 95 % CI, 2.68–285; *P* = 0.0006) was an independent predictor of postoperative cancer recurrence (Table 3). Of the components of RNS, only the L component was significantly associated with RFS rate.

**Table 3:** Cox proportional hazards analysis of R.E.N.A.L. nephrometry score and clinicopathological factors to predict recurrence-free survival

Characteristics	Univariate		Multivariate 1		Multivariate 2	
	HR (95 % CI)	*P* value	HR (95 % CI)	*P* value	HR (95 % CI)	*P* value
Age (years) (continuous)	1.04 (1.00–1.08)	0.0753	1.04 (1.00–1.08)	0.0739	1.03 (0.99–1.08)	0.0619
Gender (male vs. female)	1.34 (0.53–3.84)	0.5404				
Preoperative serum CRP level (>0.2 vs. ≤0.2 mg/dl)	2.39 (0.83–6.13)	0.0996	2.39 (0.80–6.51)	0.1114	2.82 (0.91–8.26)	0.0710
Histological grade (G1, G2 vs. G3)	1.24 (0.29–3.75)	0.7378				
Histological type (clear cell vs. non-clear cell)	1.13 (0.32–7.11)	0.8707				
Microscopic vascular involvement (+ vs. −)	2.78 (0.44–9.83)	0.2306				
R.E.N.A.L. nephrometry score sum (10–12 vs. 4–9)	7.77 (2.22–49.0)	0.0005	9.05 (2.11–63.9)	0.0019		
R.E.N.A.L. nephrometry score component						
R (3 vs. 2)	4.49 (1.77–12.8)	0.0014			2.53 (0.96–7.62)	0.0619
E (2, 3 vs. 1)	1.99 (0.72–7.00)	0.1982				
N (3 vs. 1, 2)	4.69 (0.06–401)	0.496				
L (3 vs. 1, 2)	17.49 (3.61–315)	<0.0001			15.0 (2.68–285)	0.0006
A (a vs. p)	4.48 (0.72–86.4)	0.1165	3.19 (0.48–62.7)	0.2520	2.57 (0.39–50.3)	0.3536
A (x vs. p)	4.88 (0.97–88.8)	0.0562	2.69 (0.51–49.6)	0.2859	2.15 (0.39–40.2)	0.4314
Presence of h (+ vs. −)	2.66 (1.05–7.57)	0.039	0.85 (0.30–2.74)	0.7754	0.75 (0.26–2.32)	0.6043

## Discussion

RNS was developed by Kutikov and Uzzo to standardize the assessment of anatomical features of renal tumors [1]. RNS consists of (R)adius (tumor size at maximal diameter), (E)xophytic/endophytic properties of the tumor, (N)earness of the deepest portion of tumor to the collecting system or sinus, (A)nterior (a)/posterior (p) descriptor, and the (L)ocation relative to the polar line. The suffix h (hilar) is assigned to tumors that are close to the main renal artery or vein. RNS is scored based on CT or MRI findings at diagnosis.

Because RNS was developed to quantitatively evaluate the complexity of renal tumors, many studies confirmed the usefulness of RNS for predicting perioperative outcomes in patients treated by partial nephrectomy [10–15]. Recently, some studies revealed a relationship between RNS and histological features of tumor aggressiveness [4–7]. Kutikov et al. compared the individual components of RNS with histology and grade of 525 resected tumors and constructed a novel nomogram for predicting high-grade histology. In their analyses, high R and L scores were strongly associated with high-grade histology [4]. Wang et al. validated this nomogram in 391 Chinese RCC patients [5]. Mullins et al. revealed a high RNS sum was associated with high-grade pathology in a study of 886 patients treated by robot-assisted partial nephrectomy [7].

The present study evaluated the relationship between RNS and postoperative cancer recurrence of non-small localized RCC (pT1b–pT2b). A high RNS sum was significantly associated with low RFS rate (*P* = 0.0012), and the RNS sum was a significant, independent factor for predicting postoperative cancer recurrence by multivariate analysis. Of the RNS components, only the L component was strongly associated with RFS rate. Kopp et al. revealed high RNS sum (10–12) and transfusion status were associated with shorter progression-free survival in a study of 202 patients with localized cT2 renal masses treated by radical nephrectomy or partial nephrectomy [8]. They also revealed high RNS sum, high nuclear grade, and transfusion status were associated with shorter overall survival. To date, there has been only one report suggesting that RNS was a predictive factor for postoperative cancer recurrence. The present study is the first to our knowledge that demonstrates a relationship between RNS, especially the L component, and postoperative recurrence.

Many predictive factors of recurrence after surgery in patients with RCC have been reported in the literature, and of anatomical tumor characteristics, tumor size has been identified as predictive of recurrence in many studies [16–18]. In the present study, the R component, namely, tumor size, was significantly associated with RFS rate on univariate analysis. However, upon multivariate analysis using all components of RNS, only the L component was significantly associated with RFS, whereas all other components were not significant, including R. To our knowledge, no other study has reported a similar result, making our current study unique in its findings. A study by Matsumoto et al. revealed a correlation between RNS and annual growth rates of renal masses scheduled for active surveillance [19], in which only the L component was significantly correlated with annual growth rate by multivariate analysis. These findings suggest that biological aggressiveness may be influenced by tumor location, although the underlying mechanism for this concept has not yet been fully clarified.

Among several histological characteristics, nuclear grade, presence of microvascular invasion, and tumor necrosis have been reported as predictive factors for postoperative cancer recurrence [16, 17, 20]. Of clinical and biochemical features, age at diagnosis, performance status, and the serum level of CRP have been reported as predictors of postoperative cancer recurrence [18, 20–23]. Several interesting studies have reported on molecular predictors of postoperative recurrence. Shvarts et al. reported that p53 expression in the tumor was related to nuclear grade and significantly associated with RFS rate in patients with localized RCC treated by radical nephrectomy [22]. Recently, Fujita et al. revealed that the level of vascular endothelial growth factor in preoperative serum was an independent predictor of postoperative recurrence in localized clear cell RCC [24]. Hongo et al. also revealed that cyclin-dependent kinase-related parameters were strongly associated with recurrence after surgery [25].

The present study had some limitations. First, the patient cohort of this study was small, and their T stage ranged from T1b to T2b. Considering the increasing incidence of T1a-stage tumors among RCC cases, the results of the present study may not perfectly represent localized RCC. A large study including T1a disease may produce more reliable results, but because cancer recurrence in patients with T1a RCC is rare, such a study may require a large number of patients to reach statistical significance. Second, as already mentioned, although many clinicopathological and novel molecular predictors of postoperative recurrence have been reported, our study utilized only a small number of clinicopathological parameters. Further studies would be needed to fully assess the relevance of other known predictive factors in evaluating the prognostic value of RNS on RFS rate.

In conclusion, the present study showed that RNS sum was independent predictors of postoperative recurrence in patients with non-small localized RCC (pT1b–T2b) treated by radical nephrectomy. Of the RNS components, only the L component was significantly associated with RFS. Accurate prediction of recurrence after surgical resection would be highly valuable in designing adjuvant trials, for example, in immunotherapy or molecular target therapy trials, and also for effectively scheduling follow-up visits and imaging studies.
